# Long-Term Culture of Cellular Spheroids in Novel Hydrogel Constructs for ECM Characterization in Bone Models

**DOI:** 10.3390/ma18153538

**Published:** 2025-07-28

**Authors:** Diamante Boscaro, Lill Skovholt Wahlum, Marie Eline Ullevålseter, Berit Løkensgard Strand, Pawel Sikorski

**Affiliations:** 1 Department of Physics, Faculty of Natural Sciences, Norwegian University of Science and Technology (NTNU), 7491 Trondheim, Norway; diamante.boscaro@ntnu.no (D.B.);; 2 Department of Biotechnology and Food Science, Faculty of Natural Sciences, Norwegian University of Science and Technology (NTNU), 7491 Trondheim, Norway; berit.l.strand@ntnu.no

**Keywords:** spheroids, SHIM, collagen, immunofluorescence, TEM

## Abstract

The application of cellular spheroids in bone tissue engineering research has gained significant interest in the last decade. Compared to monolayer cell cultures, the 3D architecture allows for more physiological cell–cell and cell–extracellular matrix (ECM) interactions that make cellular spheroids a suitable model system to investigate the bone ECM in vitro. The use of 3D model systems requires fine-tuning of the experimental methods used to study cell morphology, ECM deposition and mineralization, and cell–ECM interactions. In this study, we use a construct made of MC3T3-E1 cellular spheroids encapsulated in an alginate hydrogel to study and characterize the deposited ECM. Spheroid shape and structure were evaluated using confocal microscopy. The deposited collagenous ECM was characterized using Second Harmonic Imaging Microscopy (SHIM), quantitative hydroxyproline (HYP) assay, and Transmission Electron Microscopy (TEM). The use of hydrogel constructs enabled easy handling and imaging of the samples, while also helping to preserve the spheroid’s stability by preventing cells from adhering to the culture dish surface. We used a non-modified alginate hydrogel that did not facilitate cell attachment and therefore functioned as an inert encapsulating scaffold. Constructs were cultured for up to 4 weeks. SHIM, HYP assay, and TEM confirmed the deposition of a collagenous matrix. We demonstrated that alginate-encapsulated bone spheroids are a convenient and promising model for studying the bone ECM in vitro.

## 1. Introduction

To address bone-related diseases and injuries, bone tissue engineering (BTE) is currently focusing on the development of alternative cell-based methods that can overcome the limitations of traditional autologous and allogenic bone grafting [1,2]. These cell-based methods are relevant not only for regenerative applications, but also for improving understanding of bone tissue biology. One of the most significant limitations of 2D cell cultures is the inability to reproduce the in vivo microenvironment. In natural tissue, the cells are part of a complex 3D environment, in which interactions with the surrounding cells and the extracellular matrix (ECM), together with biological and mechanical stimuli, regulate cellular processes. For this reason, over the last decade, focus has been placed on the development of new cell models that can reproduce some aspects of the complex three-dimensional (3D) bone microenvironment found in vivo. To achieve this, several types of 3D in vitro cell models have been developed, such as organoids [3], organs-on-a-chip [4], and bioprinted tissues [5].

Spheroids are characterized by a spherical shape and form by spontaneous self-assembly of cells when cultured in a non-adherent environment [6]. Spheroids are extensively used in multiple fields of biomedical research due to their ability to recapitulate several aspects of natural tissues, such as the biological microenvironment [7,8,9], cell–cell and cell–ECM interactions [10,11], soluble gradients [11], and the spatial morphology of natural tissues [12]. In addition, they have shown apoptosis resistance [13,14], secretion of anti-inflammatory molecules [14,15], promotion of cell viability, protein secretion, and response to external stimuli [10,11].

The ECM is a non-cellular, complex network of fibrous proteins and glycosaminoglycans with composition dependent on the type of tissue [16,17]. The ECM properties can influence cell–cell interactions, cell proliferation, and response to external stimuli [18]. It maintains tissue stability and controls its mechanical properties; it has a role in regulating the transport of molecules and soluble factors [19], as well as being a reservoir of growth factors and ions [20]; and it is responsible for segregation between different tissues.

In bone, the ECM is involved in defining the functional properties of the tissue. The bone ECM is a complex and precisely structured inorganic–organic composite. The inorganic part consists of carbonate-substituted calcium-deficient apatite [20,21], while the organic fraction consists mainly of type I collagen (90%) and proteoglycans, as well as non-collagenous proteins (10%) such as osteocalcin and osteopontin [22]. Type I collagen is especially important as it provides mechanical stability and controls bone ECM mineralization [23]. It has a triple-helix structure (known as tropocollagen molecules), consisting of three polypeptide chains of repeating Gly-X-Y units, where X and Y are usually proline and 4-hydroxyproline, respectively [24]. The tropocollagen molecules form fibrils [23] that can interact with other proteins found in the ECM [22]. During bone mineralization, the mineral crystals will form in between and on the surface of the collagen fibers [25]. In addition, charged amino acids found in collagen bind non-collagenous proteins, which have a role in regulating mineral deposition [26].

Established methods used to investigate ECM deposition and mineralization in monolayer cell cultures need to be adapted for use in 3D cell systems, something that can be challenging for several reasons [3]. For example, limited diffusion of the dye [3] and/or attenuation of the signal in the cell aggregates [27] can limit the applicability of confocal microscopy. This can be resolved by the use of infrared radiation (IR) combined with two-photon fluorescence microscopy and Second Harmonic Imaging Microscopy (SHIM). As infrared radiation is absorbed and scattered by biological tissues to a lower extent, IR microscopy techniques are more suitable for imaging of thicker samples [28], including hydrogel-embedded spheroids. In addition, the possibility of label-free imaging of the deposited collagen matrix using SHIM makes this method an advantageous technique when performing bone mineralization studies.

In this study, spheroids embedded in an alginate hydrogel were characterized, and their ability to produce a bone-like ECM was evaluated, with a focus on collagen deposition. Confocal microscopy was used to study cell morphology and aggregate structure in intact, embedded spheroids. We focus on validation of the construct concept, comparing results between 2D and 3D, while employing multiple experimental techniques to strengthen our findings. We confirm that alginate functions as an inert carrier, with no significant interactions with encapsulated spheroids. We evaluate the ability of the embedded spheroids to produce a collagen matrix and we develop methods to characterize this matrix. The results obtained from optical microscopy and biochemical analysis are additionally supported by Transmission Electron Microscopy (TEM) images of the spheroid sections. These results demonstrate that alginate-encapsulated spheroids are a promising model system for studying bone and bone mineralization in vitro.

## 2. Materials and Methods

### 2.1. Cell Culture

MC3T3-E1 subclone 4 (ATCC, CRL-2593) was cultured in tissue culture flasks in standard conditions at 37 °C and 5% CO_2_ in minimum essential alpha medium (MEM-α without ascorbic acid; ThermoFisher (Waltham, MA, USA), A1049001) supplemented with 10% fetal bovine serum (FBS; Sigma-Aldrich (St. Louis, MO, USA)). This medium will be referred to as regular medium (RM).

To induce osteogenic differentiation, the RM was supplemented with 50 μg mL^−1^ (or 125.77 μM) of L-ascorbic acid 2-phosphate sesquimagnesium salt hydrate (Sigma-Aldrich, A8960) and 2 mM of β-glycerophosphate disodium salt pentahydrate (Sigma-Aldrich).

### 2.2. Spheroid Formation and Diameter Analysis

**Figure 1:**
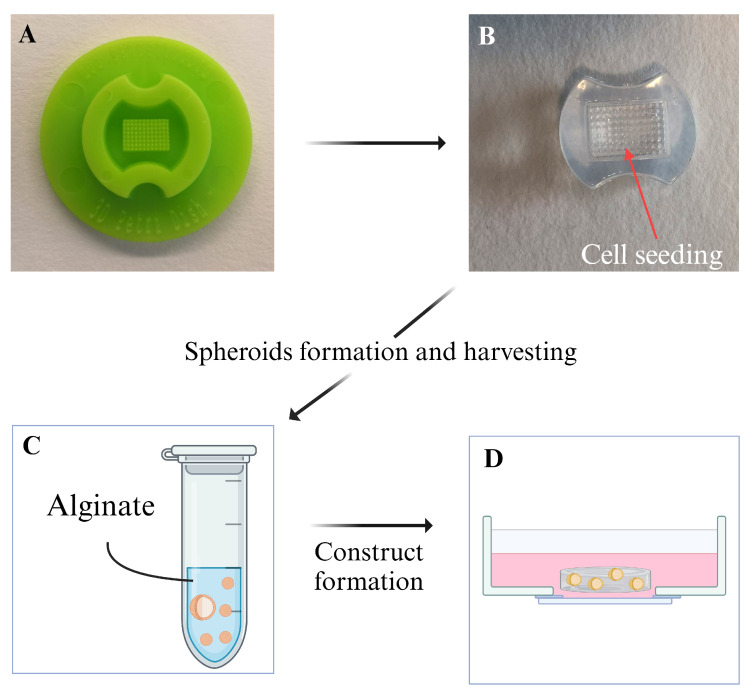
Schematic representation of spheroid formation. (**A**) The silicon mold used to create the (**B**) agarose molds. The cell suspension was placed in the central, pillared part of the mold. Spheroids were formed after ON incubation. (**C**) After collection from the molds, the spheroids were placed in a tube and resuspended in the alginate solution. (**D**) After gelation in the glass-bottomed Petri dish, the alginate disk containing the spheroids was kept in 2 mL of RM or OM.

Spheroids were obtained using the micro-mold technique [29]. Briefly, 1.5% agarose (Sigma-Aldrich) molds were made from #24-96 silicon molds (MicroTissues 3D Petri dish micro-mold spheroids, Microtissues Inc., Providence, RI, USA) (Figure 1A,B) and were placed in a 24-well plate. The cells were collected and resuspended in 1 mL of RM, and 75 μL of cell suspension was added to each mold, followed by 1 mL of RM being added to each well. After overnight incubation, the molds were placed upside down in the wells of the 24-well plate and centrifuged to allow spheroid collection. The molds were removed and the medium with spheroids was collected in a 15 mL tube and centrifuged. The sedimented spheroids were retrieved and resuspended in a solution made from an equal volume of RM and sterile filtered 2% Alginate (Alg) solution (G fraction: 0.68, GG fraction: 0.57, Molecular weight: 250,000 g mol^−1^, (Novamatrix, Sandvika, Norway) (Figure 1C). A 150 μL volume of the spheroid solution was added into glass-bottomed Petri dishes (35 mm dish with 14 mm bottom well, #1.5 glass—0.16–0.19, Cellvis, Cat. #D35-10-1.5-N), covered with a GN-6 Metricel 0.45 μm–47 mm sterile membrane, and gelation was induced by adding 50 mM CaCl_2_ on top of the membrane for 5 min. After gelation, both the CaCl_2_ solution and the membrane were removed. The alginate disks containing spheroids were kept in 2 mL of medium (Figure 1D). The medium was changed every second or third day. Spheroids were maintained under the same standard conditions as the cell cultures. An osteogenic medium (OM) was used to induce osteogenic differentiation of the spheroids in the alginate disks.

To assess their diameter, spheroids were measured on day 0 (encapsulation day), 3, and 7 using the Nikon ECLIPSE TS100 microscope (Nikon, Tokyo, Japan). Their diameter was measured using the software Fiji-ImageJ v 1.54p. The difference in diameter was compared between the RM and OM cultured spheroids.

### 2.3. Morphological and Structural Characterization

To visualize the focal adhesions in spheroids after 3 weeks of culture, the immunostaining protocol from Weiswald et al. [30] was used. This protocol was used since it ensured complete permeabilization of the spheroids, preservation of their structures, and localization of the protein of interest. Briefly, samples from the RM and OM cultures were washed with 5 mM BaCl for 5 min. Fixation and permeabilization were performed simultaneously, using 4% PFA and 1%Triton X-100 for 3 h at 4 °C. The samples were washed three times for 10 min with PBS. Samples were dehydrated in an ascending series of methanol (25%, 50%, 75%, 95%, 100%) at 4 °C in PBS and rehydrated following the same dilution series. The samples were then washed with PBS and blocked with 3% BSA + 0.1% Triton X-100 overnight at 4 °C. The following day, the samples were washed twice for 15 min, followed by incubation with the primary antibody Paxillin (5H11) Monoclonal Antibody (Invitrogen, Waltham, MA, USA, 1:200 in 1% BSA + 0.1% Triton X-100) for 48 h at 4 °C. After incubation, the samples were washed four times for 30 min. The samples were incubated with the Goat Anti-mouse IgG (H+L) Cross-Adsorbed secondary antibody Alexa Fluor^TM^ 488 (Invitrogen, 1:100 in 1% BSA + 0.1% Triton X-100) for 24 h. The following day, the samples were rinsed twice, then their nuclei were stained with Hoechst 33258 (5 μg/mL) and their cell membranes were stained with the CellMask^TM^ Deep Red plasma membrane stain (C10046) (Invitrogen, 1:1000 in PBS). The samples were then imaged using the Leica SP8 Confocal Microscope (Leica Microsystems, Wetzlar, Germany), with a HC PL Apo CS2 20×/0.75 water objective. Image analysis was performed using Fiji-ImageJ v 1.54p.

To analyse the cell morphology, spheroids cultured in RM and OM for 2 weeks were fixed and permeabilized with 4% PFA and 1% Triton X-100 in Hepes buffer for 3 h at 4 °C. The samples were then washed with Hepes buffer three times for 10 min and blocked with 3% BSA overnight. The cell actin was stained with Alexa Fluor 647 Phalloidin solution (ThermoFisher; 1:100 in Hepes for OM samples and 1:200 in Hepes for RM samples), and cell nuclei were stained with Hoechst 34580 (ThermoFisher, 1:1000 in Hepes) for 45 min at room temperature. Spheroids were imaged using a Leica SP8 Confocal Microscope, with a HC PL Apo CS2 20×/0.75 water objective. Spheroids were imaged intact, embedded in the alginate hydrogel.

To evaluate whether the reduced signal observed in the spheroids was caused by limited diffusion of the dye or by attenuation of the fluorescent signal, CellTracker Deep Red (ThermoFisher) was used to stain the cells before spheroid formation. Briefly, the cells in a tissue flask were stained with a 3 μM solution of dye and RM, and incubated for 4 h, before removing the staining solution and proceeding with spheroid formation, as described above. Imaging was performed using a CLSM Zeiss 800 Airyscan Confocal Microscope (ZEISS, Oberkochen, Germany), with a 10×/0.45 NA water objective.

### 2.4. Second Harmonic Imaging Microscopy

Collagen production in monolayer cell cultures and alginate-embedded spheroids was assessed using SHIM. Images were taken using a Leica SP8 SMD/MP Confocal Microscope, with a HCX IRAPO 25×/0.95 W (Leica Microsystems) water objective. The microscope was equipped with non-descanned detectors for detecting the SHG and two-photon excitation fluorescence signals. An 820 nm excitation was used, along with two emission filters: 390/40 nm for the Second Harmonic Generation signal and 445/20 nm for two-photon fluorescence.

Collagen production on monolayer cell cultures was assessed every week up to 4 weeks of culture in regular and osteogenic conditions. Cell were seeded in 6-well glass-bottom plates and were provided with RM and OM. At day 7, 14, 21, and 28, the cells were washed with PBS and stained for their nuclei using Hoechst 34580 (1:1000) for 20 min. After incubation, the cells were washed and imaged.

Collagen production in spheroids was assessed every week for up to 4 weeks of culture under regular and osteogenic conditions. Samples were imaged both unstained and stained for nuclei. For the stained samples, the gels were washed with PBS and stained using Hoechst 34580 (1:1000) incubated for 45 min. After incubation, the gels were washed and imaged.

The images were processed using ImageJ, and the background was subtracted to remove the signal overlap between the two channels.

Due to the partial overlap of the emission wavelengths of the collagen (410 nm) and the nuclei dye, which caused some attenuation of the signal coming from the collagen, we decided to perform imaging of unstained and stained spheroid samples. The two-photon fluorescence emission cube was also used during the imaging of the unstained samples. This overlap and attenuation effect was observed both in monolayer cell culture samples and in spheroids.

### 2.5. Transmission Electron Microscopy

For TEM analysis, 1-, 2-, and 3-week-old RM and OM cultured alginate gels with the embedded spheroids were washed with 1 mL of 5 mM BaCl, followed by fixation with 4% formaldehyde and 2.5% glutaraldehyde in PBS at 4 °C for 24 h. The fixative was removed, and the samples were cut into 1–2 mm pieces and placed in an Eppendorf tube, covered with PBS for storage. For preparation, the samples were rinsed three times in 0.1 M sodium cacodylate for 10 min, followed by post-fixation in 1% osmium tetroxide for 1 h. The samples were then rinsed three times for 10 min in water, followed by staining with 2% uranyl acetate in water for 1 h. Dehydration was performed in increasing acetone series (25 %, 50%, 75%, 90%, 96%, and 2 × 100%) for 10 min each. The samples were then embedded in a pure acetone and Epon resin mix in ratios of 2:1, 1:1, and 1:2 for 30 min each. The samples were then incubated in Epon resin, which was changed three times at intervals of 1 h, overnight, and 1 h respectively. The samples were embedded in fresh resin and polymerized at 60 °C for two days. The samples were sectioned using a Leica EM UC6 ultra-microtome at 60 nm and collected on copper TEM grids with formvar support film. Ultra-thin sections were post-stained with Reynold’s lead citrate. Spheroid sections were imaged using an Tecnai 12 (FEI Europe B.V., Eindhoven, The Netherlands) with a tungsten filament, with an acceleration voltage of 80 kV.

### 2.6. Hydroxyproline Assay

A hydroxyproline (HYP) assay was performed to assess collagen quantity in monolayer cell cultures and embedded spheroids, cultured in RM and OM. The data obtained from the HYP assay were normalized to the DNA content for each respective sample.

For 2D cell cultures, collagen quantification was performed after 1, 2, and 4 weeks of culturing in RM or OM. On the day of the analysis, the cells were washed with PBS, resuspended, and centrifuged. The cell pellets were resuspended in 200 μL of ultra-pure water. A 100 μL volume was used to perform the HYP assay, while the remaining volume was used to perform the DNA quantification.

For the spheroids, collagen quantification was performed after 1, 2, and 4 weeks of culture in RM or OM. To collect the embedded spheroids, the hydrogels were moved to a 1.5 mL Eppendorf tube and dissolved by adding 50 mM of citrate solution, then vortexed for about 5 to 10 min until gel dissolution. The gels were dissolved together to avoid sample loss. The tubes were then centrifuged at 1000 rpm for 5 min. The supernatant was removed, and the pellet was trypsinized. After resuspension in media, the samples were centrifuged, collected, and resuspended in 200 μL of ultra-pure water. A 100 μL volume was used to perform the HYP assay, while the remaining volume was used to perform the DNA quantification. The HYP assay was performed as described in [31,32]. Briefly, 100 μL of cell suspension/spheroid suspension was transferred to a pressure-tight propylene vial and 100 μL of HCl (38%) was added. The vial was capped with a Nalgene TM PPCO Low-profile Cap and was placed in an oven at 110 °C for 18 h to allow hydrolysis of collagen. The vials were then centrifuged at 10,000× *g* for 3 min and dried in the oven at 50 °C (48/72 h). After drying, samples were resuspended in 200 μL of ultra-pure water and vortexed briefly. A 60 μL volume of each sample in triplicate was transferred to a 96-well plate. Trans-4-hydroxy-L-proline was used as a standard. To each sample and standard, 20 μL of Assay Buffer (composed of 1-propanol, ultra pure water and citrate stock buffer; see Supplementary Information for more details) and 40 μL of Chloramine T reagent were added. The plate was then covered with aluminum foil and incubated for 20 min at room temperature. After the addition of 80 μL of DMBA reagent, the 96-well plate was placed in the oven at 60 °C for 60 min. The plate was left to cool down and the absorbance at 570 nm was measured using the SpectraMax i3x plate reader (Molecular Devices, San Jose, CA, USA).

DNA content was measured using the PicoGreen Quant-iT Assay Kit (Invitrogen). Cells were lysed in 0.2% Triton X-100 solution and shaken for 30 min before the DNA content measurement.

### 2.7. Statistical Analysis

The data for the monolayer cell culture represent triplicate samples derived from three independent samples. The data for the spheroids represent triplicates derived from a minimum of (independent) 2 alginate disks, each containing 10 to 25 spheroids. Data are shown as the mean value ± standard deviation. Statistical analysis was performed using one-way ANOVA with Tukey’s post-hoc test; *p* < 0.05 was considered significant. Significantly different data points are denoted with the same symbol when obtained at different time-points, and with * when comparing data from the same time-point. Non-significant differences are noted with *ns*.

## 3. Results

### 3.1. Characterization of the Embedded Spheroids: Size, Shape, Morphology, and Interaction with the Surrounding Hydrogel

**Figure 2:**
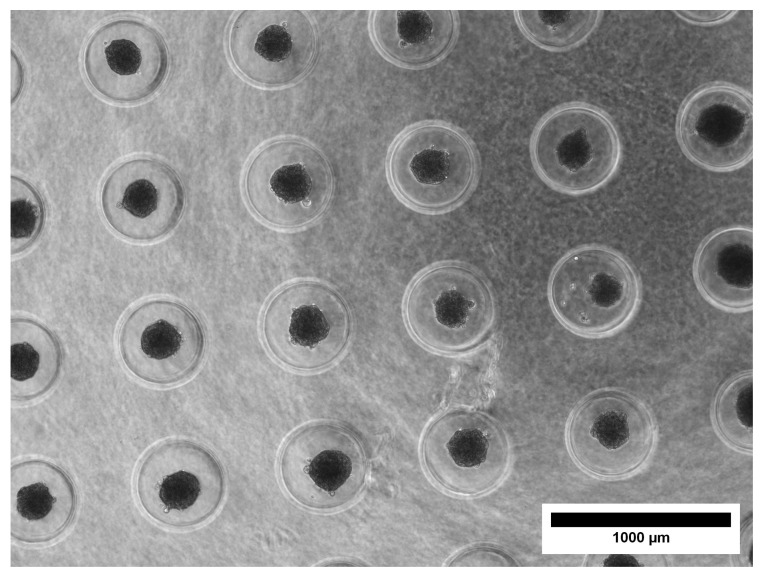
Spheroids (gray spheres) in the mold, obtained after overnight incubation.

The aim of this study was to create and characterize alginate-encapsulated spheroids with a focus on long-time culture and compatibility with microscopy and other techniques that could be used to study the produced ECM and consequently evaluate their osteogenic potential. In our model, spheroids were randomly distributed within an alginate disk that was approximately 1 mm in thickness and 5 mm in diameter, composed of 1% alginate hydrogel. The constructs were maintained in a glass-bottomed Petri dish, supplied with RM or OM. The osteogenic potential of OM-cultured spheroids was evaluated and compared to that of spheroids cultured in RM and monolayer cell cultures. Alginate was chosen for its ECM-like properties and its widespread use in the field of TE [33]. Alginate hydrogel was used as an inert scaffold that allowed for the culture and characterization of encapsulated spheroids over a prolonged culture period. Alginate hydrogel prevents cells from attaching to the culture dish surface, while alginate discs are easy to handle during the culturing period and are compatible with a variety of microscopy techniques. Spheroids from the MC3T3-E1 cell line were obtained using the micro-mold technique using agarose molds [29]. The micro-mold technique was selected for spheroid production as it allowed for easy handling of the samples during the procedure, the possibility of obtaining a relatively high number of spheroids per batch, good control over the number of cells per spheroid, and good batch-to-batch reproducibility. Spheroids in an agarose mold before harvesting are shown in Figure 2.

**Figure 3:**
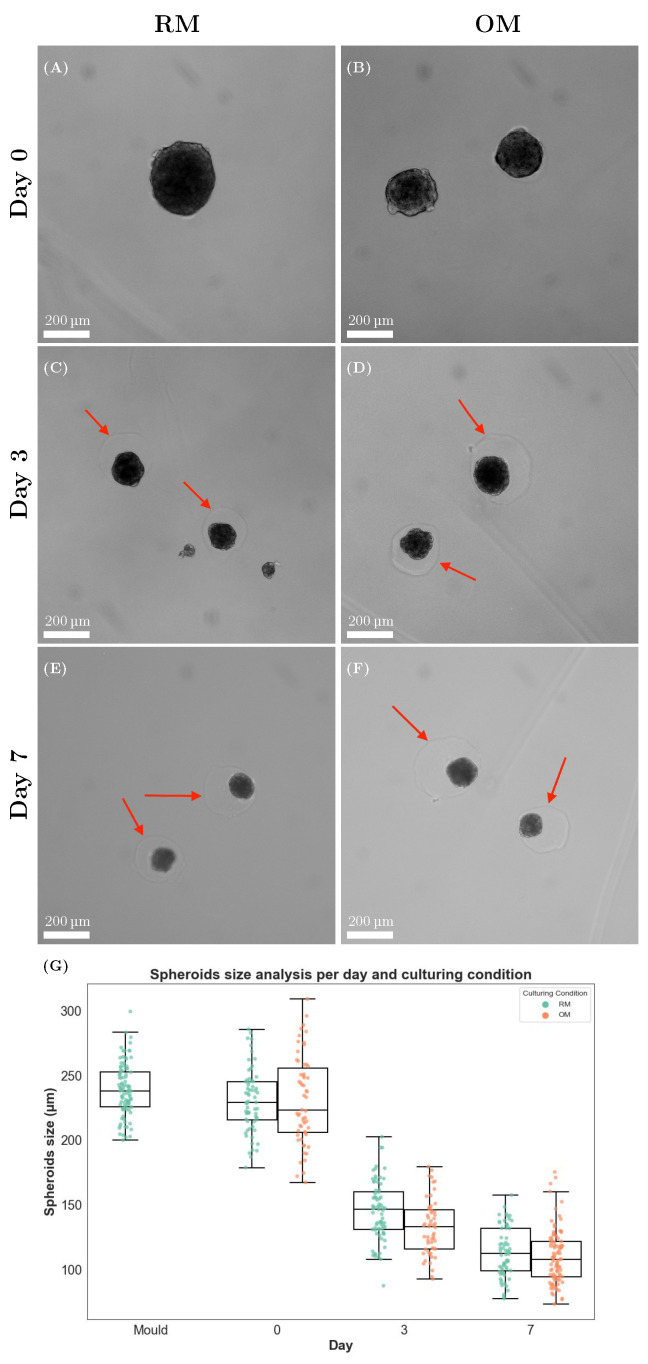
Spheroids showed a reduction in size during the first week of culture. (**A**–**F**) Bright-field microscopy images of the spheroids encapsulated in the alginate hydrogel. The red arrows indicate the outline of the pocket that was formed due to the size reduction. (**G**) Quantification of the diameter reduction of spheroids cultured in RM and OM during the first week: spheroids were measured in the mold after overnight incubation (*n* = 97), after encapsulation (day 0) in RM (*n* = 64) and OM (*n* = 58), day 3 in RM (*n* = 73) and OM (*n* = 55), and day 7 in RM (*n* = 64) and OM (*n* = 96).

Due to cell–cell interactions and the self-assembly process, spheroids were compact during the first days after preparation. This also happened when they were encapsulated in the alginate hydrogel. The spheroid size was reduced by almost 50% between day 0 and day 7, with an average diameter for RM and OM, respectively, of 230 ± 24 μm and 231 ± 34 μm on day 0 (encapsulation day), 145 ± 23 μm and 133 ± 21 μm on day 3, and 113 ± 19 μm and 110 ± 21 μm on day 7 (Figure 3A–G). No significant changes in size were observed after day 7.

As spheroids compacted, small pockets were formed in the alginate hydrogel (red arrow in Figure 3C–F). The size of these pockets was consistent with the size of the spheroids at the encapsulation time point (Day 0). For spheroids cultured for more than 1 week, small particles in the pocket area, usually close to the edges of the pocket, were observed with bright-field microscopy. These structures were identified using Transmission Electron Microscopy (TEM) as extracellular vesicles and cellular debris (Figure S1).

**Figure 4:**
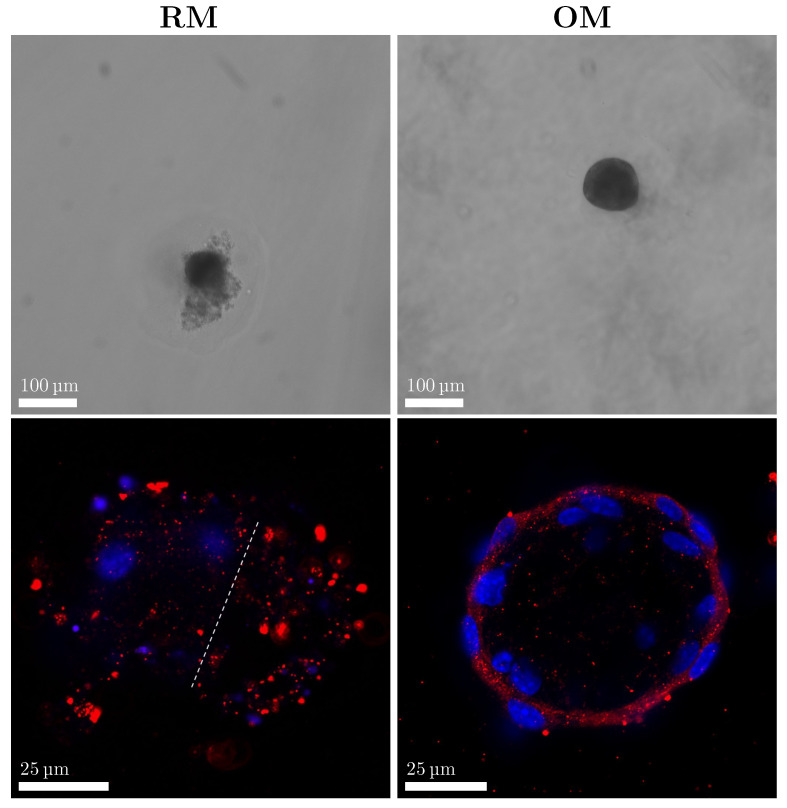
Shape analysis of spheroids cultured in RM and OM for 3 weeks. The upper images show bright-field images of the spheroids, and a difference in shape can be observed between the two different cultured samples. The lower images show immunofluorescence images of spheroids stained with nuclei (blue) and focal adhesion (red).

The shape of the spheroids were also monitored during the culturing period. The spherical shape was retained during the first 2 weeks of culture in both RM and OM. In the third week of culture, around half of the spheroids cultured in RM began to lose their spherical shape, and some protrusions were detected (Figure 4, upper left). Immunostaining for focal adhesions’ Paxillin revealed heavily stained structures in the protrusion areas (Figure 4, lower left, on the right side of the dotted white line). Figure S2 shows how these structures were also stained using a membrane dye, revealing the presence of lipidic structures in the protruding area. Major structural changes were not observed in OM-cultured spheroids, which retained their spherical shape (Figure 4, upper and lower right).

**Figure 5:**
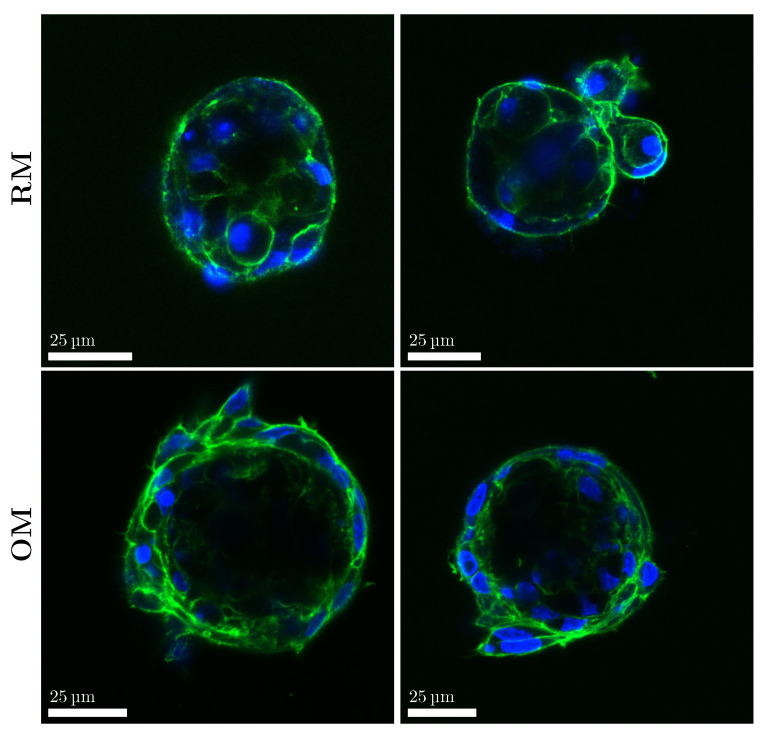
Cell morphology was influenced by the culturing conditions. Fluorescence images of actin (green) and cell nuclei (blue) of spheroids after 2 weeks in RM and OM.

To assess the response to the differentiation stimuli, we compared the morphology of the cells in spheroids cultured in RM and OM. When grown in a tissue flask, MC3T3-E1 cells in the pre-osteoblast stage were characterized by a rounder, spread-out morphology. When cultured in differentiating conditions, they showed a more elongated shape (Figure S3). To assess any change in the 3D morphology of the cells, spheroids cultured in RM and OM for 2 weeks were stained for their nuclei and actin filaments. Spheroids grown in RM displayed round nuclei and an overall round cell shape, as can be observed in the stained actin filaments (Figure 5). The cell morphology was different in the OM spheroids, where cells displayed a more elongated shape with more compacted nuclei (Figure 5).

**Figure 6:**
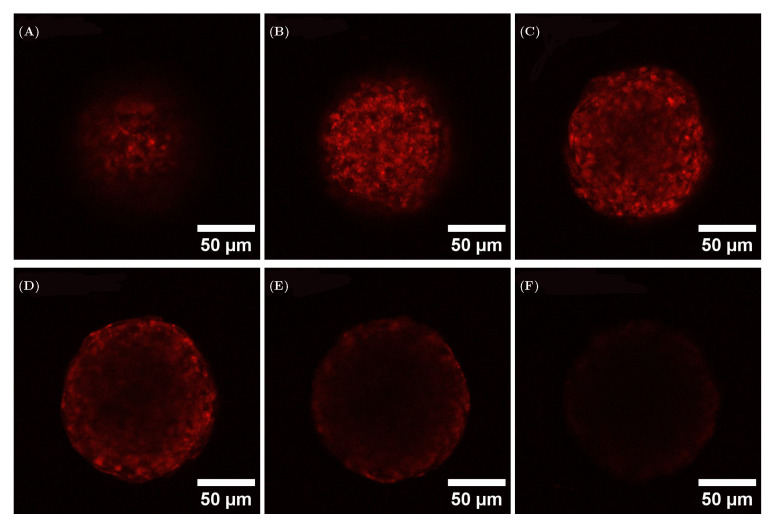
Analysis of optical effects on spheroids, treated prior to aggregation with CellTracker Deep Red. The 6 cross-sections were taken with a step of 20 μm between each other, with the first section (**A**) starting at 5 μm. (**B**) 25 μm; (**C**): 45 μm; (**D**): 65 μm; (**E**): 85 μm; (**F**): 105 μm.

During confocal microscopy imaging, a noticeably weaker fluorescence signal was observed from the core region of the spheroids. This could have resulted from restricted dye diffusion or from attenuation of the excitation or emission signals. We therefore stained cells with CellTracker Deep Red before spheroid formation, and investigated the signal intensity as a function of the position. The data obtained indicate that the loss of signal was caused by signal attenuation, as observed in images obtained at different positions along the z-axis in the same spheroid (Figure 6 and Figure S4). This result is consistent with the two-photon fluorescence microscopy results described below, where a more uniform signal from the whole aggregate was detected.

### 3.2. Osteogenic Potential of Encapsulated Spheroids

**Figure 7:**
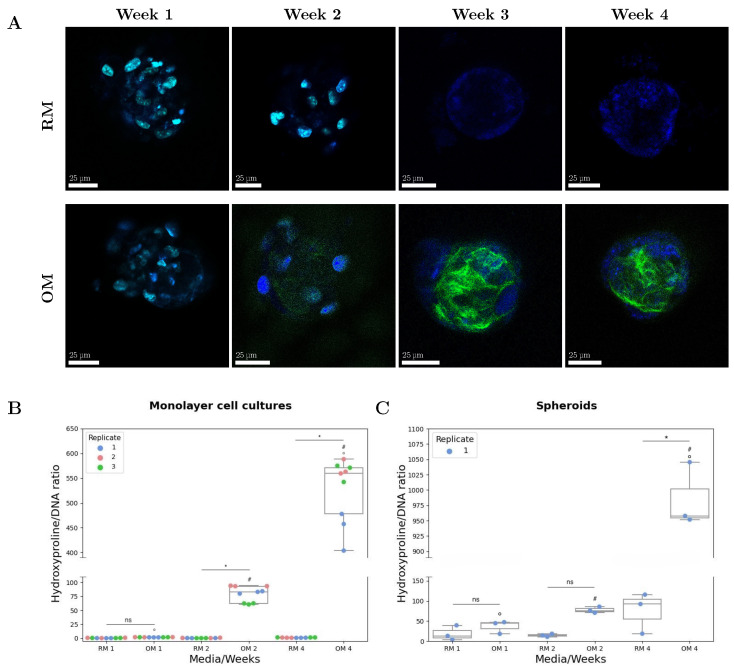
The osteogenic potential of alginate-encapsulated bone spheroids. (**A**) SHIM images of RM- and OM-cultured spheroids over 4 weeks. Collagen deposition (green) can be observed in OM-cultured spheroids at 2, 3, and 4 weeks. Spheroids cultured for 1 and 2 weeks were stained with Hoechst 34580 (blue), while spheroids cultured for 3 and 4 weeks were left unstained to ensure a better collagen signal. The blue signal observed in the unstained samples originated from 2-photon autofluorescence. Biochemical quantification of collagen in (**B**) monolayer cell cultures (n = 3) and (**C**) spheroids (n. of disks = 2). The HYP data were normalized to the DNA content. In spheroids, no statistical difference was observed between RM- and OM-cultured samples at week 1 (*p* = 0.979) and week 2 (*p* = 0.121), while a statistical difference was observed between RM- and OM-cultured samples at week 4 (*p* < 0.001). Significantly different data points are denoted with *#* and *o* for data points obtained at different time-points and with * when comparing data from the same time-point (RM v OM). Non-significant differences are noted with *ns*.

**Figure 8:**
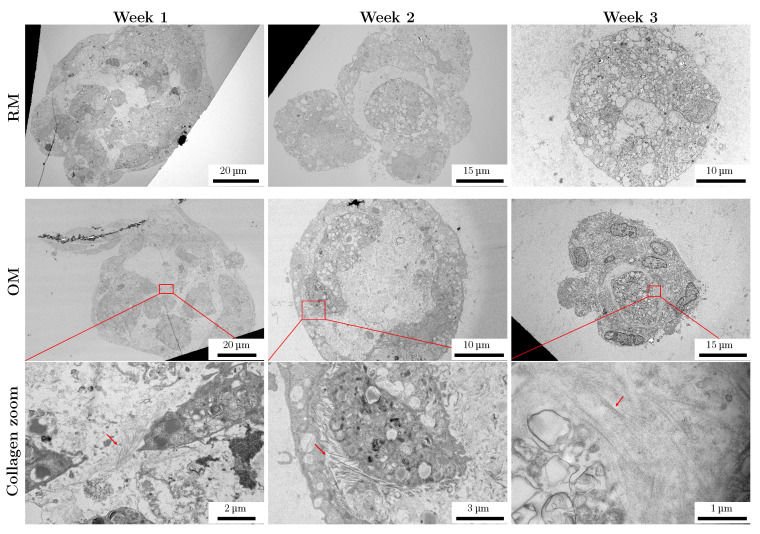
TEM images of 1-, 2-, and 3-week-old spheroids cultured in RM and OM. Regions of OM spheroids with collagen fibers are noted with a red rectangle, and a zoomed-in view of each region is shown below. Collagen fibers are indicated with red arrows.

The potential of the encapsulated spheroids to produce a bone-like matrix was investigated, with a focus on the production of collagen. Collagen deposition in spheroids in OM and RM media was assessed every week for up to 4 weeks in a qualitative manner using SHIM, and quantitatively using HYP assay. SHIM was performed on both unstained and nuclei-stained samples (Figure 7A and Figure S5). While for the stained samples (weeks 1 and 2), the nuclei are visible due to the Hoechst stain (shown in blue in Figure 7A), for the unstained samples, nuclei were detected by the two-photon auto-fluorescence signal (weeks 3 and 4, Figure 7A). Due to possible absorption of the SHG signal (410 nm) by the Hoechst dye, unstained samples showed a better signal-to-noise ratio. Images of the nuclei-stained cells are included in Figure S5. To confirm that the observed signal was originating from the deposited collagen, SHIM was also performed on monolayer cell cultures (Figure S6). The first detectable collagen deposition using SHIM was observed in spheroids cultured in OM for 14 days (Figure 7A). A progressive signal increase can be observed in the OM-cultured spheroids at week 3 and 4, corresponding to an increase in collagen deposition by the cells in the aggregates (Figure 7A), with no signal detected for RM-cultured samples at any of the time-points. No SHIM signal was observed from the packet area or the surrounding hydrogel for any of the samples examined in this study, confirming that collagen deposition was limited to the spheroids’ volume. TEM imaging of spheroids confirmed the presence of collagen fibers in samples cultured for 2 and 3 weeks in OM, but also revealed the presence of collagen in samples cultured for 1 week in OM (Figure 8, region in red rectangle). No collagen was detected in TEM images for RM-cultured spheroids (Figure 8).

Biochemical quantification of HYP confirmed the results obtained with SHIM and TEM. In particular, the HYP assay confirmed the TEM results for OM spheroids cultured for 1 week by detecting the small quantity of collagen produced by the cellular aggregates, despite the statistical analysis indicating no significant difference between the RM- and OM-cultured samples at week 1. Similarly, even though an increase in collagen content was observed at week 2 in OM-cultured samples compared to RM-cultured samples, this difference was observed to be not statistically significant (*p* = 0.121). The biochemical quantification also revealed that at the final time point (4 weeks), more collagen had been deposited by the spheroids compared to the monolayer cell cultures (Figure 7B,C) when normalized to the DNA content.

## 4. Discussion

Three-dimensional cell models, such as spheroids, more effectively replicate key aspects of natural tissues, making them a good tool for studying tissue biology [34]. Furthermore, the development of 3D constructs capable of supporting bone regeneration holds significant potential for bone tissue engineering. In these systems, it is crucial to understand the structure of the deposited extracellular matrix (ECM) and mineral phase, as well as to assess the ability of these models to accurately reproduce the natural cellular environment [35].

In this study, we investigated bone spheroids encapsulated in alginate hydrogels, with a specific focus on collagen matrix production—a fundamental step in bone tissue formation and an essential precursor for bone mineralization studies. Our long-term objective is to analyze ECM mineralization within these aggregates and characterize the deposited mineral phase.

Culturing spheroids in hydrogel discs offers a practical advantage for handling large sample quantities and facilitating different types of microscopy. In this study, spheroids were generated using MC3T3-E1 subclone 4 murine cells, a well-established model for investigating extracellular matrix (ECM) formation and mineralization [36]. As extensively documented in the literature [37,38], this cell line is easy to culture, and differentiation into osteoblast-like cells can be induced by treatment with ascorbic acid and β-glycerophosphate. For more complex bone tissue development studies, a system capable of osteocytic differentiation is required. It is known that MC3T3-E1 cells lack this ability [36]. Nonetheless, this cell line remains advantageous for short-term differentiation studies and the optimization of characterization techniques, particularly for assessing ECM production.

We observed that spheroids encapsulated in alginate disks were stable and viable, and that the alginate helped in maintaining the culture for a prolonged period. This was required for the analysis of ECM deposition and further analysis of ECM mineralization. TEM confirmed that the cells were not able to attach to or invade the alginate matrix, providing insight into the environment created in the pocket and the ECM and collagen deposited by the spheroids. Vesicles and debris in the pocket area around the spheroids could contribute to the regulation of the microenvironment and/or to cellular response during spheroid compaction and cell differentiation. Extracellular vesicles are known to be involved in cell–cell communication and signaling [39]. Entrapment of vesicles could also offer a practical way to study them after extraction from the hydrogel. For the spheroids in OM, deposition of the collagenous matrix was detected using both a quantitative assay and microscopy. The alginate hydrogel allowed for easy handling of the sample while performing microscopy analysis. The presence of the hydrogel did not influence the imaging process. Due to the nature of our sample, we decided to use SHIM as a non-invasive, label-free technique to monitor collagen deposition. To our knowledge, this method has not been used previously for the analysis of collagen in intact spheroids embedded in a hydrogel. The results obtained using this technique are in line with already-published results obtained from collagen analysis carried out using other techniques [40,41,42]. Although its sensitivity for early-stage, disorganized collagen deposition might be limited in comparison to that of TEM, SHIM remains a valuable non-destructive technique for monitoring the deposition of organized collagen fibers over time. Notably, SHIM allows for imaging of the whole aggregate, including the inner regions, something that is more challenging with confocal microscopy. In addition, due to the nature of the technique, the samples do not require preparation steps, limiting the possibility of sample damage. The possibility of analyzing living samples is also advantageous.

Collagen extraction from the alginate construct is a crucial step in implementing the quantitative collagen assay. Using a citrate solution to dissolve the alginate hydrogel has proven to be an efficient method for sample collection, minimizing sample loss. Given the relatively low cell count in 3D spheroid samples, a highly sensitive assay is necessary to quantitatively detect produced collagen. The HYP assay employed in this study demonstrated sufficient sensitivity, enabling the detection of collagen produced by approximately 30 spheroids at each time point and for each culture condition, with each spheroid containing around 3000 to 3500 cells. The higher HYP/DNA ratio observed in spheroids compared to monolayer cell cultures at 4 weeks suggests that the 3D microenvironment may positively influence the osteogenic differentiation and promote collagen deposition. This difference is not related to the confluency of 2D cultures, as cells reach confluency after approximately 10 days, and the increase in collagen deposition was observed up to 4 weeks.

Our observations show that the deposited collagenous ECM plays a crucial role in maintaining spheroid stability and shape. After approximately 3 weeks of culture, RM-cultured spheroids began to lose their spherical structure and show ECM proteins, and cells became dispersed within less organized regions. These protrusions originated from potentially proliferating cells that detached from the outer layer. The focal adhesion immunostaining performed on the RM cultured samples revealed the presence of stained structures in the protrusions. In contrast, OM-cultured spheroids remained structurally stable, likely due to the significant collagen matrix that supported aggregate integrity from week 2 in culture. These findings suggest that cells in the outer layer may exhibit some proliferative capacity, though the extent of cell proliferation within the aggregate remains a topic of ongoing debate [11,13,43].

## 5. Conclusions

We have demonstrated that alginate-encapsulated bone spheroids are a promising model to study the bone ECM in vitro. We have verified the construct concept, demonstrating that spheroids can be successfully maintained in culture in alginate disks for at least four weeks in both osteogenic and growth conditions. Collagen production in 3D cell cultures was verified and systematically compared with that in 2D cell cultures, using both a hydroxyproline (HYP) assay and microscopy. We have developed an extraction protocol enabling precise collagen quantification that is compatible with the alginate matrix. To further characterize the system, we employed immunostaining and Transmission Electron Microscopy (TEM) to visualize focal adhesions and the molecular organization of collagen at different time points. Our results highlight the critical role of the ECM in spheroid stability during long-term culture, as only spheroids in which the cells produced an ECM were stable over the culture period. SHIM proved to be an effective method for monitoring the collagen matrix in intact and encapsulated spheroids, while two-photon fluorescence microscopy enhanced the visualization of structures such as cell nuclei within the spheroids, benefiting from the improved signal penetration depth of the IR excitation laser.

## Data Availability

The original contributions presented in this study are included in the article/Supplementary Material. Further inquiries can be directed to the corresponding author.

## Supplementary Materials

The following supporting information can be downloaded at https://www.mdpi.com/article/10.3390/ma18153538/s1: Figure S1: TEM image of the vesicles released in the pocket. The edge of the spheroid can be seen at the bottom of the figure, while the extracellular vesicles and the cellular debries can be seen as the small, darker, spherical structures. These vesicles are usually observed to be closer to the edge of the pocket. Their presence show and confirm that no interaction is happening between the spheroid and the hydrogel; Figure S2: Shape analysis of 3 weeks RM (left) and OM (right) cultured spheroids. Nuclei are shown in blue, focal adhesions in red and cell membranes in green. It can be noticed that the protrusion area in the RM cultured spheroids (right side of the dotted white line) was stained for both focal adhesions and cell membrane; Figure S3: Evaluation of cell morphology in monolayer cells cultured in RM and OM. Figure A shows cells cultured in RM and Figure B shows cells cultured in OM. Cells seeded in RM show a more spread shape compared to OM cultured cells, characterized by a more elongated morphology. The same characteristic was observed in cells making up the spheroid; Figure S4: Intensity profile collected from RM cultured spheroid, 2 days after encapsulation, using CLSM. Results were plotted as intensity variations as a function of the distance along the positive x-axis, where x is the diameter of the spheroid along the x-axis (138 µm). The interval between each section is 20 µm, with a total Z-stack height of 135 µm. The data demonstrates that the fluorescence signal is significantly attenuated by the dense cell aggregate. Original data points are shown as dashed lines and solid lines represent interpolated data; Figure S5: Collagen deposition in spheroids. 2 weeks old samples were left unstained for the nuclei, while 3 and 4 weeks old samples were stained for the nuclei (blue). The blue signal coming from the unstained samples resulted to be auto-fluorescence from the cells. Collagen fibers can be seen in green. A strong signal attenuation can be observed when comparing stained and unstained samples at the same time point; Figure S6: Collagen deposition in monolayer cell cultures. Cells were cultured in RM and OM for 4 weeks and were imaged for collagen production every week up to 4 weeks. The images show nuclei (blue) and collagen (green). A progressive increase in the presence of collagen can be observed starting from week 1, where small and localized spots of deposited collagen were observed, to week 4, where a uniform layer of collagen lays on top of the cells. No collagen deposition was observed in RM cultured samples.

## References

[1] Agarwal R., García A.J. (2015). Biomaterial strategies for engineering implants for enhanced osseointegration and bone repair. Adv. Drug Deliv. Rev..

[2] Amini A.R., Laurencin C.T., Nukavarapu S.P. (2012). Bone Tissue Engineering: Recent Advances and Challenges. Crit. Rev. Biomed. Eng..

[3] Urzì O., Gasparro R., Costanzo E., De Luca A., Giavaresi G., Fontana S., Alessandro R. (2023). Three-Dimensional Cell Cultures: The Bridge between In Vitro and In Vivo Models. Int. J. Mol. Sci..

[4] Huh D., Hamilton G.A., Ingber D.E. (2011). From 3D cell culture to organs-on-chips. Trends Cell Biol..

[5] Liu S., Cheng L., Liu Y., Zhang H., Song Y., Park J.H., Dashnyam K., Lee J.H., Khalak F.A.H., Riester O. (2023). 3D Bioprinting tissue analogs: Current development and translational implications. J. Tissue Eng..

[6] Kim W., Gwon Y., Park S., Kim H., Kim J. (2023). Therapeutic strategies of three-dimensional stem cell spheroids and organoids for tissue repair and regeneration. Bioact. Mater..

[7] Baptista L., Kronemberger G., Côrtes I., Charelli L., Matsui R., Palhares T., Sohier J., Rossi A., Granjeiro J. (2018). Adult Stem Cells Spheroids to Optimize Cell Colonization in Scaffolds for Cartilage and Bone Tissue Engineering. Int. J. Mol. Sci..

[8] Bellotti C., Duchi S., Bevilacqua A., Lucarelli E., Piccinini F. (2016). Long term morphological characterization of mesenchymal stromal cells 3D spheroids built with a rapid method based on entry-level equipment. Cytotechnology.

[9] Imamura A., Kajiya H., Fujisaki S., Maeshiba M., Yanagi T., Kojima H., Ohno J. (2020). Three-dimensional spheroids of mesenchymal stem/stromal cells promote osteogenesis by activating stemness and Wnt/β-catenin. Biochem. Biophys. Res. Commun..

[10] Gionet-Gonzales M.A., Leach J.K. (2018). Engineering principles for guiding spheroid function in the regeneration of bone, cartilage, and skin. Biomed. Mater..

[11] Kim S., Kim E.M., Yamamoto M., Park H., Shin H. (2020). Engineering Multi-Cellular Spheroids for Tissue Engineering and Regenerative Medicine. Adv. Healthc. Mater..

[12] Li C., Zhang Y., Du Y., Hou Z., Zhang Y., Cui W., Chen W. (2023). A Review of Advanced Biomaterials and Cells for the Production of Bone Organoid. Small Sci..

[13] Decarli M.C., Amaral R., Santos D.P.d., Tofani L.B., Katayama E., Rezende R.A., Silva J.V.L.d., Swiech K., Suazo C.A.T., Mota C. (2021). Cell spheroids as a versatile research platform: Formation mechanisms, high throughput production, characterization and applications. Biofabrication.

[14] Jensen C., Teng Y. (2020). Is It Time to Start Transitioning from 2D to 3D Cell Culture?. Front. Mol. Biosci..

[15] Laschke M.W., Menger M.D. (2017). Life is 3D: Boosting Spheroid Function for Tissue Engineering. Trends Biotechnol..

[16] Padhi A., Nain A.S. (2020). ECM in Differentiation: A Review of Matrix Structure, Composition and Mechanical Properties. Ann. Biomed. Eng..

[17] Hoshiba T., Chen G., Endo C., Maruyama H., Wakui M., Nemoto E., Kawazoe N., Tanaka M. (2016). Decellularized Extracellular Matrix as an In Vitro Model to Study the Comprehensive Roles of the ECM in Stem Cell Differentiation. Stem Cells Int..

[18] Chen S., Chen X., Geng Z., Su J. (2022). The horizon of bone organoid: A perspective on construction and application. Bioact. Mater..

[19] Saraswathibhatla A., Indana D., Chaudhuri O. (2023). Cell–extracellular matrix mechanotransduction in 3D. Nat. Rev. Mol. Cell Biol..

[20] Mansour A., Mezour M.A., Badran Z., Tamimi F. (2017). Extracellular Matrices for Bone Regeneration: A Literature Review. Tissue Eng. Part A.

[21] Clarke B. (2008). Normal Bone Anatomy and Physiology. Clin. J. Am. Soc. Nephrol..

[22] Lin X., Patil S., Gao Y.G., Qian A. (2020). The Bone Extracellular Matrix in Bone Formation and Regeneration. Front. Pharmacol..

[23] Selvaraj V., Sekaran S., Dhanasekaran A., Warrier S. (2024). Type 1 collagen: Synthesis, structure and key functions in bone mineralization. Differentiation.

[24] Oosterlaken B.M., Vena M.P., De With G. (2021). In Vitro Mineralization of Collagen. Adv. Mater..

[25] Abou Neel E., Aljabo A., Strange A., Ibrahim S., Coathup M., Young A., Bozec L., Mudera V. (2016). Demineralization & remineralization dynamics in teeth and bone. Int. J. Nanomed..

[26] Yu L., Wei M. (2021). Biomineralization of Collagen-Based Materials for Hard Tissue Repair. Int. J. Mol. Sci..

[27] Tibbitt M.W., Anseth K.S. (2009). Hydrogels as extracellular matrix mimics for 3D cell culture. Biotechnol. Bioeng..

[28] Campagnola P. (2011). Second Harmonic Generation Imaging Microscopy: Applications to Diseases Diagnostics. Anal. Chem..

[29] Napolitano A.P., Dean D.M., Man A.J., Youssef J., Ho D.N., Rago A.P., Lech M.P., Morgan J.R. (2007). Scaffold-free three-dimensional cell culture utilizing micromolded nonadhesive hydrogels. BioTechniques.

[30] Weiswald L.B., Guinebretière J.M., Richon S., Bellet D., Saubaméa B., Dangles-Marie V. (2010). In situ protein expression in tumour spheres: Development of an immunostaining protocol for confocal microscopy. BMC Cancer.

[31] Hollander A.P., Hatton P.V. (2003). Biopolymer Methods in Tissue Engineering.

[32] Ignat’eva N.Y., Danilov N.A., Averkiev S.V., Obrezkova M.V., Lunin V.V., Sobol’ E.N. (2007). Determination of hydroxyproline in tissues and the evaluation of the collagen content of the tissues. J. Anal. Chem..

[33] Lee K.Y., Mooney D.J. (2012). Alginate: Properties and biomedical applications. Prog. Polym. Sci..

[34] Pandit A., Indurkar A., Locs J., Haugen H.J., Loca D. (2024). Calcium Phosphates: A Key to Next-Generation In Vitro Bone Modeling. Adv. Healthc. Mater..

[35] De Pace R., Iaquinta M.R., Benkhalqui A., D’Agostino A., Trevisiol L., Nocini R., Mazziotta C., Rotondo J.C., Bononi I., Tognon M. (2025). Revolutionizing bone healing: The role of 3D models. Cell Regen..

[36] Mertz E.L., Makareeva E., Mirigian L.S., Leikin S. (2023). Bone Formation in 2D Culture of Primary Cells. JBMR Plus.

[37] Brochado A.C.B., Silva D.C., Silva J.C.d., Lowenstein A., Gameiro V.S., Mavropoulos E., Mourão C.F., Alves G.G. (2023). Characterization and Applicability of a Bone Spheroid Model for the Evaluation of Cytocompatibility of Bone Substitutes. Appl. Sci..

[38] Addison W., Nelea V., Chicatun F., Chien Y.C., Tran-Khanh N., Buschmann M., Nazhat S., Kaartinen M., Vali H., Tecklenburg M. (2015). Extracellular matrix mineralization in murine MC3T3-E1 osteoblast cultures: An ultrastructural, compositional and comparative analysis with mouse bone. Bone.

[39] Van Niel G., Carter D.R.F., Clayton A., Lambert D.W., Raposo G., Vader P. (2022). Challenges and directions in studying cell–cell communication by extracellular vesicles. Nat. Rev. Mol. Cell Biol..

[40] Koblenzer M., Weiler M., Fragoulis A., Rütten S., Pufe T., Jahr H. (2022). Physiological Mineralization during In Vitro Osteogenesis in a Biomimetic Spheroid Culture Model. Cells.

[41] Gonzalez-Fernandez T., Tenorio A.J., Saiz A.M., Leach J.K. (2022). Engineered Cell-Secreted Extracellular Matrix Modulates Cell Spheroid Mechanosensing and Amplifies Their Response to Inductive Cues for the Formation of Mineralized Tissues. Adv. Healthc. Mater..

[42] Sanchez A.A., Teixeira F.C., Casademunt P., Beeren I., Moroni L., Mota C. (2025). Enhanced osteogenic differentiation in hyaluronic acid methacrylate (HAMA) matrix: A comparative study of hPDC and hBMSC spheroids for bone tissue engineering. Biofabrication.

[43] Kim J., Adachi T. (2019). Cell Condensation Triggers the Differentiation of Osteoblast Precursor Cells to Osteocyte-Like Cells. Front. Bioeng. Biotechnol..

